# Self-rated treatment outcomes in medical rehabilitation among German and non-German nationals residing in Germany: an exploratory cross-sectional study

**DOI:** 10.1186/s12913-016-1348-z

**Published:** 2016-03-28

**Authors:** P. Brzoska, O. Sauzet, Y. Yilmaz-Aslan, T. Widera, O. Razum

**Affiliations:** Department of Epidemiology & International Public Health, Bielefeld University, School of Public Health, Bielefeld, Germany; Chemnitz University of Technology, Faculty of Behavioral and Social Sciences, Institute of Sociology, Chemnitz, Germany; Department of Public Health, Giresun University, Faculty of Medicine, Giresun, Turkey; German Statutory Pension Insurance Scheme, Social Medicine and Rehabilitation, Section Rehabilitation Research (Deutsche Rentenversicherung Bund, Geschäftsbereich Sozialmedizin und Rehabilitation, Bereich Reha-Wissenschaften), Berlin, Germany

**Keywords:** Migrants, Inequity, Rehabilitation, Effectiveness, Health services research

## Abstract

**Background:**

In many European countries, foreign nationals experience, on average, less favorable treatment outcomes in rehabilitative care than the respective majority population. In Germany, this for example is reflected in a lower occupational performance and a higher risk of disability retirement after rehabilitation as analyses of routine data show. However, little is known about the perspective of health care users. The aim of the present study was to compare self-rated treatment outcomes between German and non-German nationals undergoing in-patient medical rehabilitation in Germany.

**Methods:**

We analyzed data from a cross-sectional representative rehabilitation patient survey of 239,811 patients from 642 clinics in Germany who completed about 3 weeks of in-patient rehabilitative treatment. The self-rating of the treatment outcome was based on a dichotomized Likert scale consisting of three items. A multilevel logistic regression analysis adjusted for various demographic, socio-economic, health and other covariates was conducted to examine differences in the self-rated treatment outcome between German and non-German nationals.

**Results:**

Of the 239,811 respondents 0.9 % were nationals from Turkey, 0.8 % had a nationality from a former Yugoslavian country, 0.9 % held a nationality from the South European countries Portugal, Spain, Italy or Greece and 1.9 % were nationals from other countries. Non-German nationals reported a less favorable self-rated outcome than Germans. Adjusted odds ratios [OR] for reporting a less favorable treatment outcome were 1.24 (95 %-confidence interval [95 %-CI]: 1.12–1.37) for nationals from the South European countries Portugal/Spain/Italy/Greece, 1.62 (95 %-CI: 1.45–1.80) for Turkish nationals and 1.68 (95 %-CI: 1.52–1.85) for nationals from Former Yugoslavia.

**Conclusions:**

Knowledge on health outcomes from the patients’ point of view is important for the provision of patient-centered health care. Our study showed that non-German nationals report less favorable outcomes of rehabilitative care than Germans. This may be due to cultural and religious needs not sufficiently addressed by health care providers. In order to improve rehabilitative care for non-German nationals, rehabilitative services must become sensitive to the needs of this population group. Diversity management can contribute to this process.

## Background

Foreign nationals comprise increasingly large proportions of the populations in many European countries. They differ in some health-related aspects from the majority populations of the countries they reside in. This comprises patterns of disease, health outcomes and health behavior [1, 2]. In Germany, 8.6 % of the total population (equaling about 6.8 million individuals) has a non-German citizenship according to the microcensus, a representative household survey in Germany [3]. Nationals from Turkey, Former Yugoslavia and the South European countries Portugal, Spain, Italy and Greece constitute about half of the population of non-German nationals residing in Germany. Many of these individuals or their parents were recruited as labor migrants between the 1950s and the 1970s and then settled in the country followed by their families [3].

Data on the health of migrants is limited [4]. The few available studies show a multifaceted picture. On the one hand, non-Germans have some health advantages over the majority population, e.g., with respect to a lower incidence of certain types of cancer. On the other hand, they have, on average, a higher prevalence of work-related disability and of chronic disorders such as diabetes mellitus type 2. They also experience higher rates of occupational accidents and occupational diseases [2, 5]. There is evidence that these differences are due to various environmental and social factors which migrants are exposed to in different phases of their life, comprising the time before, during and after the migration process. Negative exposures in the host country include, amongst others, poor working conditions, a lower socio-economic status and a poor language proficiency [2, 6].

The German health care system is ethically, legally and socially responsible for providing adequate health services to the entire population of their countries—including residents with a foreign nationality [4]. Rehabilitative services are of particular relevance for non-German nationals considering their overall higher burden of chronic diseases and higher rates of work-related disability as outlined above. As a measure of tertiary prevention, rehabilitation can mitigate the consequences of acute and chronic diseases and can restore a deteriorated health status [7].

In Germany, rehabilitative care is covered by different institutions of the health care system. The German Statutory Pension Insurance Scheme covers rehabilitative treatments for individuals in working age as well as for patients with cancer. Together, they make up for about two-thirds of all cases seeking rehabilitative care in Germany [8]. The rehabilitative services as covered by the German Statutory Pension Insurance Scheme are usually provided as 3-weeks in-patient programs conducted in specialized clinics [9]. Rehabilitative services for people in retirement age with diseases other than cancer are covered by other social security institutions, mainly by the Statutory Health Insurance [8].

Only few studies have been carried out on the effectiveness of rehabilitation in Germany. Many of them support the effectiveness of rehabilitative treatments but are usually restricted to particular clinical conditions [10]. The scarce evidence is critically appraised by the Advisory Council on the Assessment of Developments in the Health Care System in its recent report and reveals future research need in this field [10].

Non-Germans and Germans have equal rights to use rehabilitation free of charge as part of their social insurance (the situation is different for asylum seekers who are initially only entitled to receive emergency care for acute health problems free of charge [2]). However, studies based on routine and survey data show that non-Germans utilize rehabilitation less often than Germans [11–13]. They also experience, on average, less favorable treatment outcomes, which is reflected in a lower occupational performance and a higher risk of disability retirement after rehabilitation [11, 13–15]. This is particularly true for individuals with a nationality from Turkey and Former Yugoslavia. Differences in the utilization and outcomes of rehabilitation between German and non-German nationals cannot be explained by differences in health status or demographic and socio-economic factors [11, 13–15]. Similar results were reported from other countries [16–19]. For example, Sloots et al. observed higher rates of drop-out from rehabilitation among Turkish and Moroccan migrants as compared to the Dutch majority population in the Netherlands [16, 17].

One important limitation of previous investigations into the rehabilitative care of migrants in Germany is that little is known about the perspective of health care users. Subjective treatment effects perceived by patients such as quality of life and self-rated performance, however, are important indicators of health care quality and essential patient-reported outcomes [20, 21]. They predict objective treatment outcomes such as return to work and prevention of early retirement [22–24] and are also strongly correlated with doctors’ evaluation of clinical outcomes [25]. Considering patients’ point of view, for example by evaluating self-rated treatment outcomes, is immanent to the provision of patient-centered health care and to the achievement of outcomes that are relevant to patients [26, 27]. In Germany, several investigations in the rehabilitation setting have shown that self-rated treatment outcomes are associated with demographic and socio-economic factors, and that they may vary between patients with different clinical conditions [28–34].

Little is known about the self-rated treatment outcomes in rehabilitative services among migrants. Possible difference in these outcomes between migrants and non-migrants after adjusting for common social determinants could be indicative of health disparities and could contribute to improving rehabilitative care for this population group. Gruner et al. studied a small sample of migrants and non-migrants in Germany who underwent in-patient medical rehabilitation for psychosomatic conditions [35]. They showed that migrants tended to have a less favorable self-rated work-related performance after rehabilitation than non-migrants. The study, however, was conducted in only one clinic in South Germany. Because of its exploratory character, differences between migrants and non-migrants in terms of health status as well as demographic and socio-economic characteristics could not be adjusted for. Hence, it remains unclear whether differences in the self-rated outcome merely reflect the influence of confounding variables or point towards inequities in health care between both populations.

The aim of the present study was to address these limitations by means of a large sample which allows to compare self-rated treatment outcomes between Germans and non-Germans undergoing in-patient medical rehabilitation in all regions of Germany. Furthermore, the sample allowed us to take different covariates into account which might confound the relationship between nationality and outcomes of rehabilitative care.

## Methods

### Data

We analyzed data from a cross-sectional representative rehabilitation patient survey implemented by the German Statutory Pension Insurance Scheme (‘Deutsche Rentenversicherung’) among individuals who completed about 3 weeks of in-patient rehabilitative care granted by this institution. Rehabilitative treatments for people in retirement age with diseases other than cancer are covered by other social security institutions such as the Statutory Health Insurance are not included in our analysis.

The patient survey is part of an external quality assurance program implemented by the German Statutory Pension Insurance Scheme for its rehabilitation clinics following legal requirement as defined by the German Social Code VI and IX. From each rehabilitation facility, 20 patients who completed medical rehabilitation are randomly selected on a monthly basis and approached at home 8–12 weeks after their treatment by means of a postal self-administered questionnaire. They provide a written informed consent regarding participation in the survey. The survey is conducted in German language. The average response rate per year is 55 % [36]. The collection and use of the data for research purposes (secondary data analysis) is in accord with the legal requirements for routinely assessed data as defined by the German Social Code X. Also, the data are fully anonymized. Therefore, no additional ethical approval for the current analysis was necessary [37].

For the present study, we drew on data from 274,513 individuals diagnosed with somatic conditions who underwent in-patient medical rehabilitation in one of 624 rehabilitation clinics during 2007–2011.

### Measures

As part of the rehabilitation patient survey, patients are asked to assess the degree to which the rehabilitative service they utilized improved (a) their health status, (b) their occupational performance and (c) their performance in everyday life and leisure-time activities. This can be considered a relative assessment because it is based on a subjective comparison of the perceived health status before and after utilization of the service. The assessment of each of these three items is conducted on a five-point Likert scale (1=“considerably deteriorated”, 2=“slightly deteriorated”, 3=“unchanged”, 4 = “slightly improved”, 5=“considerably improved”). A correlation analysis carried out for the present study showed that the three items were highly correlated ranging from *r* = 0.7–0.8 (Spearman rho). A mean score based on the responses to the three items was calculated and dichotomized (<4 “perceived health/performance deteriorated or unchanged” vs. ≥4 “perceived health/performance improved”).

We were able to compare Germans with four groups of non-German nationals: Turkey, Former Yugoslavia, Portugal/Spain/Italy/Greece and ‘other’, similar to previous studies based on routine data of the German Statutory Pension Insurance Scheme [11, 14]. Information on nationality included in the routine data of the German Statutory Pension Insurance Scheme is obtained from official registry data [37].

In line with previous research we also controlled for relevant covariates which have been shown to be associated with treatment outcomes [28–34]. These covariates are based on a covariate model suggested for analyses of routine data of the German Statutory Pension Insurance Scheme [33]. These covariates were *age* (in years), *sex*, *marital status* (single/divorced/widowed, married), *education* (low, intermediate, high, other/unknown) *occupational position* (skilled labor, semi-skilled/unskilled labor, trainee/unemployed), the *type of somatic diagnosis* on admission to rehabilitation (diseases of the skeletal system, neoplasms, diseases of the circulatory system, other), the *time absent from work due to illness in the last 12 months before rehabilitation* (0 months, <3 months, 3 to <6 months, ≥6 months, not employed) and the *perceived occupational performance before rehabilitation* (low, moderate/high, not mentioned). The latter two variables were considered as proxies for disease severity before rehabilitation. Furthermore, following procedures in other studies [33, 38–40], information on whether respondents received *assistance in completing the self-administered questionnaire* was considered as a proxy for German language proficiency, for comprehensibility of the questionnaire and for other factors that limited patients in filling in the questionnaire on their own. Finally, the *overall satisfaction* of the patient with the care received (low/moderate, high) and *type of rehabilitation* (rehabilitation directly following a hospital stay vs. [optional] rehabilitation provided independently of a prior hospital stay) were accounted for. Since early retirement in Germany cannot be granted for individuals unless their potential for rehabilitation is fully exhausted, seeking optional rehabilitative treatment may be indicative of a high motivation for early retirement and a low motivation for a successful completion of rehabilitation [41]. All variables had less than 3 % of missing values.

### Statistical analysis

Tabulation stratified by nationality was used for sample description. Given the large sample size no significance tests were conducted for this purpose. Multilevel logistic regression models were used to analyze the relationship between nationality (independent variable) and the perceived treatment outcome (dependent variable). To adjust for covariates, multivariable models were computed. The multilevel analysis was performed to account for the clustering of respondents within the 642 rehabilitation clinics. The intraclass correlation coefficient (ICC) was calculated following the multivariable analysis to estimate the proportion of the total variance in the perceived treatment outcome that is due to differences between clinics rather than differences between individuals [42].

For all models, odds ratios (OR) and 95 % confidence intervals (95 %-CI) were calculated. All analyses were performed using the software *Stata*, version 12 [43].

## Results

Data for 239,811 individuals was available with complete information for all variables. Of these, 0.9 % (*n* = 2155) were nationals from Turkey, 0.8 % (*n* = 2267) had a nationality from a former Yugoslavian country, 0.9 % (*n* = 2061) held a nationality from the South European countries Portugal, Spain, Italy or Greece and 1.9 % (*n* = 4846) were nationals from other countries. In total, 4.5 % (*n* = 11,329) of all respondents participating in the survey had a non-German nationality.

Table 1 shows the characteristics of the study sample stratified by nationality (group). German and non-German nationals differed in their demographic and socio-economic characteristics. Amongst others, this is reflected in a higher proportion of non-German respondents who were male, who only had a low school education and who worked in semi-skilled/unskilled occupational positions. Differences also become evident with respect to underlying diseases conditions, with larger proportions of non-Germans compared to Germans, for instance, undergoing rehabilitation because of diseases of the skeletal system. Furthermore, the proportions of patients who reported a moderate/high self-rated performance before rehabilitation was lower among non-Germans than among Germans. The same applied to the proportion of individuals who were highly satisfied with the rehabilitative care received.

**Table 1:** Description of the study sample stratified by nationality (participants of the rehabilitation client survey of the German Statutory Pension Insurance Scheme conducted between 2007 and 2011, *n* = 251,140)

	Nationality										Total (*n* = 251,140)	
	Germany (*n* = 239,811)		Turkey (*n* = 2,155)		Former Yugoslavia (*n* = 2,267)		Portugal/Spain/Italy/Greece (*n* = 2,061)		Other (*n* = 4,846)			
Age in years (mean; SD)												
	53.3	10.0	47.8	9.4	53.2	8.8	52.1	8.8	52.0	8.9	53.2	10.0
Sex (n, %)												
Male	119,434	49.8	1,361	63.2	1,216	53.6	1,331	64.6	2,666	55.0	126,008	50.2
Female	120,377	50.2	794	36.8	1,051	46.4	730	35.4	2,180	45.0	125,132	49.8
Marriage status (n, %)												
Single/divorced/widowed	67,364	28.1	266	12.3	463	20.4	410	19.9	1,145	23.6	69,648	27.7
Married	172,447	71.9	1,889	87.7	1,804	79.6	1,651	80.1	3,701	76.4	181,492	72.3
School education (n, %)												
Low	105,407	44.0	1,526	70.8	1,235	54.5	1,471	71.4	1,644	33.9	111,283	44.3
Intermediate	77,195	32.2	286	13.3	514	22.7	264	12.8	1,199	24.7	79,458	31.6
High	35,491	14.8	106	4.9	243	10.7	136	6.6	1,149	23.7	37,125	14.8
Other/unknown	21,718	9.1	237	11.0	275	12.1	190	9.2	854	17.6	23,274	9.3
Occupational position (n, %)												
Skilled labor	167,134	69.7	866	40.2	1,060	46.8	963	46.7	2,729	56.3	172,752	68.8
Semi-skilled/unskilled labor	32,368	13.5	1,093	50.7	992	43.8	918	44.5	1,583	32.7	36,954	14.7
Trainee/not employed	40,309	16.8	196	9.1	215	9.5	180	8.7	534	11.0	41,434	16.5
Time absent from work in the last 12 months (n, %)												
None	36,920	15.4	285	13.2	249	11.0	237	11.5	722	14.9	38,413	15.3
< 3 months	112,589	46.9	895	41.5	1,036	45.7	1,008	48.9	2,249	46.4	117,777	46.9
3–6 months	25,381	10.6	348	16.1	359	15.8	291	14.1	637	13.1	27,016	10.8
6+ months	28,809	12.0	445	20.6	420	18.5	346	16.8	770	15.9	30,790	12.3
not employed	36,112	15.1	182	8.4	203	9.0	179	8.7	468	9.7	37,144	14.8
Diagnosis at rehabilitation entry (n, %)												
Skeletal system	103,986	43.4	1,184	54.9	1,248	55.1	1,052	51.0	2,299	47.4	109,769	43.7
Neoplasms	46,810	19.5	155	7.2	306	13.5	280	13.6	669	13.8	48,220	19.2
Circulatory system	32,633	13.6	286	13.3	261	11.5	271	13.1	678	14.0	34,129	13.6
Respiratory system	8,438	3.5	66	3.1	41	1.8	69	3.3	175	3.6	8,789	3.5
Other	47,944	20.0	464	21.5	411	18.1	389	18.9	1,025	21.2	50,233	20.0
Type of rehabilitation (n, %)												
Hospital follow-up	87,735	36.6	728	33.8	719	31.7	697	33.8	1,737	35.8	91,616	36.5
Not hospital follow-up	152,076	63.4	1,427	66.2	1,548	68.3	1,364	66.2	3,109	64.2	159,524	63.5
Assistance received in completing questionnaire (n, %)												
No	228,963	95.5	937	43.5	1,413	62.3	1,112	54	3,613	74.6	236,038	94
Yes	10,848	4.5	1,218	56.5	854	37.7	949	46	1,233	25.4	15,102	6
Overall satisfaction (n, %)												
High	183,709	76.6	1,244	57.7	1,575	69.5	1,434	69.6	3,715	76.7	191,677	76.3
Low/moderate	56,102	23.4	911	42.3	692	30.5	627	30.4	1,131	23.3	59,463	23.7
Self-rated performance before rehabilitation (n, %)												
Moderate/high	98,567	37.5	679	31.5	693	30.6	733	35.6	1,806	37.3	93,939	37.4
Low	90,028	41.1	1,087	50.4	1,187	52.4	974	47.3	2,184	45.1	103,999	41.4
Not stated	51,216	21.4	389	18.1	387	17.1	354	17.2	856	17.7	53,202	21.2
Subjective treatment outcome (n, %)												
Health/performance improved	129,861	54.2	605	28.1	781	34.5	784	38.0	2,401	49.5	134,432	53.5
Health/performance deteriorated or unchanged	109,950	45.8	1,550	71.9	1,486	65.5	1,277	62.0	2,445	50.5	116,708	46.5

*Note. SD* standard deviation

As is further shown in Table 1, the percentage of respondents who reported that their health/performance improved through their rehabilitative treatment varied between the different groups of non-German nationals. While 54.2 % of all Germans reported an improvement of their health/performance following rehabilitation, the respective proportions were considerably lower for non-German nationals, being 28.1 % for Turkish nationals, 34.5 % for Former Yugoslavian nationals and 38.0 % for nationals from Portugal, Spain, Italy or Greece.

Around 5 % of the total variance in the perceived treatment outcome can be explained by the 642 rehabilitation clinics (ICC = 0.046), warranting a multilevel approach to represent heterogeneity in the data. The results of the multilevel regression model with deteriorated or unchanged health/performance as the dependent variable adjusted for covariates are displayed in Table 2. It becomes evident that the differences in the perceived treatment outcome between the nationality strata still exist even after controlling for demographic and socioeconomic characteristics as well as health and other covariates. As compared to German nationals, Turkish and Former Yugoslavian nationals had a 62 and 68 % higher chance, respectively, of an unfavorable perceived treatment outcome (OR = 1.62; 95 %-CI: 1.45–1.80 and OR = 1.68; 95 %-CI: 1.52–1.85, respectively). The respective chance among nationals from Portugal, Spain, Italy or Greece was 24 % higher (OR = 1.24; 95 %-CI: 1.12–1.37).

**Table 2:** Results of the multivariable logistic regression model with deteriorated or unchanged health/performance as the dependent variable. Odds ratios (OR) and 95 % confidence intervals [95 %-CI] (participants of the rehabilitation client survey of the German Statutory Pension Insurance Scheme conducted between 2007 and 2011, *n* = 251,140)

	OR	95 %-CI
Nationality		
Germany (Reference)		
Portugal/Spain/Italy/Greece	1.24	(1.12; 1.37)
Former Yugoslavia	1.68	(1.52; 1.85)
Turkey	1.62	(1.45; 1.80)
Other	1.08	(1.01; 1.15)
*Age in years*	1.02	(1.01; 1.02)
Sex		
Male (Reference)		
Female	0.90	(0.88; 0.92)
Marriage status		
Single/divorced/widowed (Reference)		
Married	0.93	(0.91; 0.94)
School education		
High (Reference)		
Intermediate	1.92	(1.87; 1.98)
Low	1.44	(1.40; 1.48)
Other/unknown	1.46	(1.40; 1.51)
Occupational position		
Skilled labor (Reference)		
Semi-skilled/unskilled labor	1.29	(1.26; 1.33)
Trainee/not employed	1.15	(1.09; 1.21)
Time absent from work in the last 12 months		
None (Reference)		
< 3 months	0.98	(0.95; 1.01)
3–6 months	1.32	(1.28; 1.37)
6+ months	2.01	(1.94; 2.08)
not employed	1.08	(1.01; 1.14)
Diagnosis at rehabilitation entry		
Skeletal system (Reference)		
Neoplasms	0.94	(0.90; 0.99)
Circulatory system	1.12	(1.07; 1.18)
Respiratory system	0.95	(0.88; 1.03)
Other	1.04	(1.01; 1.08)
Type of rehabilitation		
Hospital follow-up (Reference)		
Not hospital follow-up	1.22	(1.19; 1.25)
Assistance received in completing questionnaire		
No (Reference)		
Yes	1.72	(1.65; 1.79)
Overall satisfaction		
High (Reference)		
Low/moderate	7.65	(7.47; 7.84)
Self-rated performance before rehabilitation		
Mediocre/high (Reference)		
Low	1.46	(1.43; 1.49)
Not stated	1.81	(1.74; 1.87)

*Note. OR* odds ratio, *95 %-CI* 95 % confidence interval

Aside from non-German nationality, a low or intermediate educational status and occupational position, a longer time absent from work in the last 12 months before rehabilitation, a low self-rated performance before rehabilitation and a low/moderate satisfaction with the rehabilitation service, amongst others, were associated with a higher chance of a poor perceived outcome of rehabilitative care.

## Discussion

In many European countries, foreign nationals benefit less from health care services than the respective majority populations. In Germany, this is particularly reflected in (tertiary) preventive services such as rehabilitation. Considering the overall higher burden of chronic diseases and impairment among non-German nationals, rehabilitative services are of particular importance. Knowledge on self-rated treatment outcomes can help to improve health care for this population group and to reduce existing health disparities.

The aim of the present investigation was to compare self-rated treatment outcomes in rehabilitation between German and non-German nationals residing in Germany. It shows that non-German nationals report a less favorable outcome of their rehabilitative treatment than Germans. A previous study, which showed that migrants undergoing psychosomatic rehabilitation have a poorer self-rated work performance after the treatment than non-migrants, was limited in two important respects. First, it was based on a small sample from one clinic in Germany. Second, it was exploratory and was not able to take differences between migrants and non-migrants in terms of health status as well as demographic and socio-economic characteristics into account [35]. By means of representative data for Germany covering all somatic rehabilitations through the years 2007–2011, we add to these findings by illustrating that differences in the self-rated treatment outcomes cannot be explained by demographic and socio-economic factors or a different disease profile and health status before rehabilitation alone. Since we adjusted for the satisfaction with health care, it is also unlikely that poorer perceived health outcomes are merely a reflection of dissatisfaction with treatment which has been observed among migrants [44, 45]. Our results are also in accord with previous research. It showed that the association between non-German nationality and objective outcomes of rehabilitative care such as the occupational performance as evaluated by health professionals [11, 15] or the risk of disability retirement after rehabilitation [14] cannot be explained by a different distribution of demographic, socio-economic and health variables between the two population groups. These studies, as does the present investigation, show that patients of Turkish and Former Yugoslavian origin tended to be more vulnerable to a poor treatment outcome than patients originating from the South-European countries Portugal, Spain, Italy or Greece.

One possible explanation for our findings may be various barriers that migrants experience in the health care system. Several qualitative investigations in somatic health care in general [46, 47] as well as rehabilitation in particular [48, 49] have highlighted different challenges and obstacles migrants face in the German health care setting. They interfere with an adequate provision of care and may result in reduced health care quality. These challenges result from a low German language proficiency which is still prevalent among many, particularly older, migrants residing in Germany [50]. In rehabilitation this can lead to communication problems between patients and health care providers by making it difficult for health professionals to obtain the medical history of patients or to instruct patients about therapies and necessary exercises. A low proficiency in the language of the host country may also negatively affect health literacy [51]. Health literacy is an important determinant of informed decision making in health care [52] and has been shown to be limited among migrant populations residing in Germany [53, 54]. For the present study it must be considered, however, that the rehabilitation patient survey is conducted in German language which is why only patients with a sufficient amount of German language proficiency or who receive assistance in completing the questionnaire take part in the survey. Since we adjusted for help received in the completion of the questionnaire, low language proficiency cannot fully explain the findings of our investigation. Aside from problems due to poor language proficiency also cultural and religious needs, expectations and preferences (e.g., with respect to illness perceptions, the way symptoms and illness are expressed, coping with disease or cultural and social taboos), which are not sufficiently accounted for by health care institutions may affect the patient-provider relationship negatively when inadequately addressed in the health care process. Similar to low German language proficiency they can lead to conflicts between health care users and providers and may reduce health care quality [19, 20].

The association between an unfavorable perceived outcome of rehabilitation and the covariates which we took into account in our multivariable analysis to adjust for potential confounding are mostly in line with those identified in other studies on treatment outcomes in rehabilitation [11, 14, 15, 55–57]. In our study, some of the covariates had a larger effect size than nationality. Still, despite adjusting for these variables, considerable differences between the nationality strata existed.

### Strengths and limitation

A strength of our study is the use of data from a representative rehabilitation patient survey which is routinely conducted by a large social security institution in Germany covering the majority of rehabilitative treatments in the country [8]. Given different measures of quality control implemented by this institution, the quality of the data can be considered high [37]. A previous investigation into the perceived treatment outcomes in rehabilitation [35] was limited by its narrow focus on one clinic and a small sample size which only allowed a descriptive analysis. By means of a representative survey we were able to give an account of rehabilitative treatments by the German Statutory Pension Insurance Scheme provided in the entire country and to control for different covariates potentially obscuring the relationship between nationality and outcomes of rehabilitative care [11].

Our study also has some limitations. The rehabilitation patient survey is conducted in German language. Although we tried to control for a possible bias resulting from low German language proficiency by adjusting the multivariable model for assistance that survey participants received when completing the questionnaire, it is likely that some patients with little German language proficiency did not take part in the survey (information on non-responders are not available). This may have introduced a selection bias, which in turn may explain why the proportion of non-German (particularly Turkish) nationals in the total sample of respondents was lower than could have been expected based on routine data on all completed rehabilitations covered by the German Statutory Pension Insurance Scheme. This data shows that about 1.4, 1.1 and 1.0 % of all patients who completed a rehabilitative treatment in the years 2007–2011 had a nationality from Turkey, Former Yugoslavia and Portugal/Spain/Italy/Greece, respectively. Given proportions of 0.8, 0.9 and 0.9 % of nationals from Turkey, Former Yugoslavia and Portugal/Spain/Italy/Greece, respectively, who took part in the patient survey, it becomes evident that the difference between the expected and observed proportion of non-German individuals in our sample is particularly pronounced for Turkish patients. Proportions for nationals from Former Yugoslavia and Portugal/Spain/Italy/Greece only differ slightly. A reason for this could be that Turkish migrants in Germany are particularly prone to low German language proficiency [58]. Based on studies which compared rehabilitative outcomes between German and non-German nationals by means of routine data on all completed rehabilitations covered by the German Statutory Pension Insurance Scheme, it can be assumed that the differences between German nationals and non-Germans in our study would be even more pronounced if a multilingual survey would have been used. Since, to our knowledge, no studies have been conducted on differences in rehabilitative outcomes between individuals with poor and good German language proficiency which would allow to quantify a potential selection bias, this issue should be subject of future research.

Because of the limitations inherent to the data set (and to many similar routine data sets in Germany [59]) we were only able to define migrants through a non-German nationality. Since other information which would allow to assess migration status more thoroughly such as the country of origin or the birth place of parents are missing, only a part of the population of migrants could be studied in our investigation. We consider the resulting bias to be small because other studies in the field that were able to also consider migrants of German nationality (for example those who immigrated to Germany and acquired German citizenship) did not identify relevant differences between this population and non-Germans in terms of outcomes of rehabilitative care [15, 60].

Patient surveys may be prone to a recall bias, particularly when patients are asked to evaluate a clinic stay that took place several months ago [61]. In the present case, we do not consider this to have an impact on our results as the main focus of our investigation is the effectiveness of rehabilitation as it is rated by patients already 6 weeks after rehabilitation and with respect to activities at work and everyday life. In addition, there is no reason why recall bias should differ between the population groups. Our data, though, only allows a cross-sectional analysis with patients evaluating the outcome of rehabilitation 8–12 weeks after discharge. It is possible that the self-rated effectiveness of rehabilitation as well as differences between the population groups change over time [61].

We used information on whether respondents received assistance in completing the self-administered questionnaire as a proxy measure for factors limiting patients in filling in the survey instrument on their own. This procedure is in line with other studies [38–40] and aims to control for a potential bias arising from the assisted completion. However, no further information on the type of limiting factors are available, which may, amongst others, include poor language proficiency but also the inability to read and write because of impairment.

As mentioned previously, our study only covers somatic in-patient rehabilitations provided by the German Statutory Pension Insurance Scheme. Further research needs to investigate whether our results can also be extended to out-patient rehabilitative services, to services targeting mental conditions and to services provided by other social security institutions in Germany.

Finally, our study is based on cross-sectional data. However, we used a type of outcome measure which allows an indirect adjustment for baseline health differences. Thus, we consider the difference in the self-rated treatment outcome that we identified between the population groups very informative and indicative of health disparities. Still, more experimental studies on the effectiveness of rehabilitation in Germany are urgently needed [10].

## Conclusions

Knowledge on health outcomes from the patients’ point of view is important for health care providers so they can provide services to users which meet their subjective and objective needs. Our study showed that non-German nationals report less favorable outcomes of rehabilitative care. This may be due to cultural and religious needs not sufficiently addressed by health care providers. Different strategies are available to address cultural and religious heterogeneity in health care and to provide services catering for the diversity of patients [62–64]. They comprise cross-cultural trainings for health providers as well as the employment of health navigators and interpreters. Another more holistic approach is diversity management which not only allows to take into account the needs of migrants but which also acknowledges that expectations and preferences in health care do not only vary with migration status or culture but also with other diversity characteristics such as sex, age and socioeconomic position [65]. Diversity management comprises different measures. They include, for example, drafting diversity-sensitive mission statements, identifying strengths and limitations related to the diversity of patients and staff, promoting self-reflection and an open attitude, as well using pictographic information systems in health facilities to allow an easy way of orientation for patients with limited reading capabilities [66–68].

Implementing diversity management in health care institutions can improve health care for the entire population as has been shown by evaluation studies [69, 70]. As stated by the Advisory Council on the Assessment of Developments in the Health Care System for Germany, more studies need to be conducted on the effectiveness of rehabilitation in Germany [10]. While observational studies such as the present one provide valuable insights into determinants of favorable treatment outcomes, also experimental studies employing a longitudinal approach need to be implemented. Since these studies usually require substantial resources, sufficient funding by stakeholders in the German system of rehabilitation must be provided.

## Abbreviations

OR: odds ratio
95 %-CI: 95 %-confidence interval
ICC: Intraclass correlation coefficient
